# The Role of Volatile Organic Compounds and Rhizosphere Competence in Mode of Action of the Non-pathogenic *Fusarium oxysporum* FO12 Toward Verticillium Wilt

**DOI:** 10.3389/fmicb.2019.01808

**Published:** 2019-08-02

**Authors:** Antonio Mulero-Aparicio, Tomislav Cernava, David Turrà, Angelika Schaefer, Antonio Di Pietro, Francisco Javier López-Escudero, Antonio Trapero, Gabriele Berg

**Affiliations:** ^1^Grupo de Patología Agroforestal, Escuela Técnica Superior de Ingenieros Agrónomos y de Montes, Departamento de Agronomía, Universidad de Córdoba, Córdoba, Spain; ^2^Institute of Environmental Biotechnology, Graz University of Technology, Graz, Austria; ^3^Departamento de Genética, Campus de Excelencia Internacional Agroalimentario (ceiA3), Universidad de Córdoba, Córdoba, Spain

**Keywords:** anti-fungal volatiles, biological control, mode of action, Verticillium wilt, mVOCs

## Abstract

Verticillium wilts caused by *Verticillium* spp. are among the most challenging plant diseases to control and affect numerous hosts worldwide. Due to the lack of effective, conventional control methods, integrated control strategies provide a promising approach to manage these diseases. The non-pathogenic *Fusarium oxysporum* strain FO12 was reported in previous studies to be an effective biocontrol agent against *Verticillium dahliae*, however, its mode of action remains to be elucidated. In this study, complementary *in vitro* and *in vivo* experiments were conducted in order to explore the implications of inhibitory substances and rhizosphere competence in antagonistic effects of FO12 against *V. dahliae* and *V. longisporum*. Volatile organic compounds and soluble substances produced by FO12, which caused significant inhibition of mycelial growth and microsclerotia viability in the two tested *Verticillium* species, were identified by means of gas and liquid chromatography-mass spectrometry. We showed that the antagonistic effect of *F. oxysporum* FO12 is partially due to the production of bioactive compounds such as 3-methyl-1-butanol and 2-methyl-1-butanol, among others. Several metabolic pathways of FO12 were altered upon contact with *V. dahliae* ELV22 volatiles. The reduced production of alpha, alpha-trehalose, a metabolite used in starch and sucrose metabolism, suggests that the biocontrol agent activates its stress response in the presence of the phytopathogen. Microscopic analysis using sGFP-tagged FO12 on oil seed rape as a model plant suggests that the biocontrol strain is an efficient root colonizer, which could compete with *V. dahliae* in the same ecological niche. The findings obtained in this study provide new insights into the mode of action of this potential biocontrol agent, which are relevant for controlling Verticillium wilt through an ecologically friendly approach.

## Introduction

*Verticillium* species are generally widely distributed in soil, and are common plant endophytes (Pegg and Brady, 2002; Barbara and Clewes, 2003; Klosterman et al., 2009). However, distinct *Verticillium* species represent a devastating group of plant pathogens that cause wilt disease in a large number of hosts worldwide. Representative species within this genus such as *Verticillium albo-atrum* (Reinke and Berthold, 1879), *V. dahliae* Kleb. (1913), and *V. longisporum* C. Stark (Karapapa et al., 1997) among others (Inderbitzin et al., 2011), are commonly found in agricultural soils. However, their abundance was drastically enhanced in various plant cultivation areas due to short crop rotations and monocultures in intense agriculture. Increasing soil temperatures due to global warming further aggravate their capacity to infect host plants (Tjamos et al., 2000). Moreover, changes in their genomes resulting from inter-kingdom horizontal events have enhanced their adaptability and pathogenicity (Shi-Kunne et al., 2019). Currently, these pathogenic species cause losses in many herbaceous and woody crops with important economic impact (Hiemstra, 1998; Pegg and Brady, 2002). While *V. dahliae* and *V. albo-atrum* can infect a high number of host species, *V. longisporum* has a more limited host range, primarily infesting *Brassicaceae* crops (Daebeler et al., 1988; Zeise and von Tiedemann, 2002; Depotter et al., 2016). Verticillium wilts caused by *V. dahliae* have a high economic impact causing severe yield losses crops such as cotton and olive in temperate and subtropical regions (Pegg and Brady, 2002). During recent years, Verticillium wilt has become a major challenge for olive growing in the Mediterranean basin countries, due to the lack of an effective control method (López-Escudero and Mercado-Blanco, 2011). In Spain, a disease occurrence of 39% in affected orchards was reported in the last decades (Blanco-López et al., 1984; Sánchez-Hernández et al., 1998).

The control of Verticillium wilts is one of the most difficult challenges for growers due to the broad range of hosts that can be colonized by the pathogens. Other aggravating factors are the location of the pathogen within the xylem vessels of the infected plants, the long-lasting viability of their microsclerotia (resting structures), the genetically heterogeneous and polyphyletic character of *Verticillium* isolates and the lack of effective fungicide treatments, among others (Fradin et al., 2009; López-Escudero and Mercado-Blanco, 2011; Jiménez-Gasco et al., 2014). However, the dispersal, incidence and severity of Verticillium wilts can be partially reduced by means of integrated disease management and enhanced biodiversity (López-Escudero and Mercado-Blanco, 2011; Berg et al., 2017). In this context and due to the increased concern about environmental and human health, the use of eco-friendly alternatives such as biological control measures, have become potential tools to improve the efficiency of integrated disease management (Berg, 2009; Berg et al., 2017). These approaches are perceived as safe and have a minimal environmental impact. Several studies have reported the use of BCAs such as *Serratia plymuthica* HRO-C48 (Müller and Berg, 2008), non-pathogenic *Verticillium* strains (Tyvaert et al., 2014) or strains of *Paenibacillus* and *Serratia* (Kurze et al., 2001; Rybakova et al., 2016) against *V. longisporum*. Likewise, studies conducted during the last 15 years have reported the use of different antagonistic microorganisms as BCAs against *V. dahliae* in herbaceous and horticultural crops such as oilseed rape, tomato, pepper or cotton (Tjamos et al., 2004; Xue et al., 2013; Rybakova et al., 2016; Veloso et al., 2016). The most studied BCAs against *V. dahliae* in olive are *S. plymuthica* (Müller et al., 2008), *Paenibacillus alvei* (Markakis et al., 2016), *Pseudomonas* spp. (Mercado-Blanco et al., 2004; Triki et al., 2012) and *Trichoderma* spp. (Jiménez-Díaz et al., 2009).

Recently, a large-scale screening of potentially beneficial microorganisms for the biocontrol of VWO yielded a non-pathogenic *Fusarium oxysporum* isolate (FO12) as one of the most effective BCAs against the pathogen (Varo et al., 2016b). However, there is no knowledge related to the underlying antagonistic effects of non-pathogenic strains of *F. oxysporum* against *V. longisporum*. In contrast, several studies on the interaction between non-pathogenic *F. oxysporum* strains and *V. dahliae* have been performed in herbaceous crops, with promising results (Pantelides et al., 2009; Angelopoulou et al., 2014; Veloso et al., 2016). Previous studies suggest that non-pathogenic isolates of *F. oxysporum* have different modes of action (Fravel et al., 2003), including competition, antibiosis and/or induction of systemic resistance in plants (Pantelides et al., 2009; Zhang et al., 2015; Veloso et al., 2016). One specific mode of action reported for some non-pathogenic strains of *F. oxysporum* is the production of VOCs with antifungal activity against pathogenic *formae speciales* of *F. oxysporum* (Minerdi et al., 2009) and against *V. dahliae* in cotton (Zhang et al., 2015). A major advantage of VOCs when compared to larger molecules is their capacity to diffuse over large distances. Cumulative data suggest that volatiles play a more important role for microbial interactions than non-volatile substances (Kanchiswamy et al., 2015). Various studies have demonstrated that microbial volatiles can significantly reduce the viability and proliferation of devastating plant pathogens such as *Botrytis cinerea*, *F. oxysporum* or *Magnaporthe oryzae* (Minerdi et al., 2009; Cernava et al., 2015a). Moreover, it was shown that exchange of aerial signals such as VOCs between microorganisms can induce a change in the recipient’s metabolism (Rybakova et al., 2017). This response can enhance or reduce the production of specific soluble metabolites to guarantee the recipient’s survival in the environment. The mode of interaction is often strain-specific; therefore, a detailed understanding of the specific mode of action of a BCA is crucial for the development of an efficient biocontrol strategy.

The objective of this study was to contribute to the understanding of the mode of action of non-pathogenic *F. oxysporum* FO12 toward pathogenic Verticillium species in the rhizosphere. FO12 was able to reduce the mycelial growth of the phytopathogenic *V. dahliae*, the viability of its microsclerotia in naturally infested soils and demonstrated a significant reduction of VWO in *in vivo* experiments (Varo et al., 2016b). Therefore, we elucidate the modes of action by (i) testing the effect of VOCs produced by FO12 on mycelial growth and microsclerotia viability of *V. dahliae* and *V. longisporum*; (ii) identifying the chemical nature of the VOCs produced by FO12; (iii) assessing changes in the metabolism of FO12 after exposure to *V. dahliae* VOCs; and (iv) studying root colonization by FO12 in a model plant by means of CLSM.

## Materials and Methods

### Fungal Strains and Growth Conditions

The fungal pathogens used in this study were *V. longisporum* (C. Stark) (Karapapa et al., 1997) strain ELV25 and *V. dahliae* Kleb. strains ELV22, V004 and V024. The strains ELV22 and ELV25 from the collection of the Institute of Environmental Biotechnology (Graz University of Technology), were described by Messner et al. (1996). The mild-virulent strain V004 was classified as non-defoliating pathotype (Blanco-López et al., 1989), and the high-virulent strain V024 was classified as defoliating pathotype (Varo et al., 2016b). Both were obtained from the fungal collection of the Agronomy Dpt. of the University of Córdoba. The non-pathogenic *F. oxysporum* strain FO12, also from the fungal collection of the Agronomy Dpt. of the University of Córdoba, was applied as BCA. Single-spore cultures of all isolates were prepared prior to use by means of the serial dilution method and maintained on potato dextrose agar (PDA; Difco^®^ Laboratories, MD, United States) slants at 4°C. 7-day-old single spore cultures incubated on PDA at room temperature were used as an inoculum source.

### Generation of sGFP-Tagged *F. oxysporum* FO12 Transformants

Green fluorescent protein-labeled strains of FO12 were obtained by co-transforming fungal protoplasts with the hygromycin resistance and the sGFP expression cassette, as previously described (Di Pietro et al., 2001; López-Berges et al., 2012). Cytoplasmic sGFP expression was analyzed in at least twenty independent transformants using a Zeiss Axio Imager M2 microscope (Zeiss, Barcelona, Spain) equipped with a GFP (BP 450/490, FT 510, LP 515) filter set and an Evolve Photometrics EM512 digital camera (Photometrics Technology, Tucson, AZ, United States). Transformants exhibiting the highest sGFP expression and retaining efficient biocontrol activity against *Verticillium* isolates were used in subsequent live-cell microscopy studies.

### Effects of Volatile Metabolites From *F. oxysporum* FO12 Against *V. longisporum* and *V. dahliae*

The antagonistic effect of the VOCs produced by *F. oxysporum* FO12 and by the transformant FO12-sGFP against all *Verticillium* isolates used in this study was tested by means of the “Two Clamp VOCs Assay” as described in Cernava et al. (2015a). Mycelial plugs (3 mm ø) of each isolate were obtained from the margin of 7-day-old colonies grown on PDA as described above. For each *Verticillium* isolate, a mycelial plug was placed in the center of the wells of a 6-well plate (Greiner Bio-One, Frickenhausen, Germany) previously filled with 3 mL of PDA per well. Subsequently, one mycelial plug of *F. oxysporum* isolate FO12 and FO12-sGFP was transferred to the same position of a 6-well plate placed opposite to the plate with the pathogen. A perforated (0.5 cm ø) 1mm silicone foil was placed between both 6-well plates for tightening connected wells and usual clamps for fixation. Additionally, six wells with plugs of the pathogens connected to a plate only with PDA were used as a control. Plates with ELV25 and ELV22 and with V004 and V024 were incubated at room temperature for 4 and 5 days, respectively. The assay was performed in six replicates (six wells for each *Verticillium* isolate, FO12 strain, and control combination) randomly distributed in three 6-well plates (two wells per treatment and plate) to avoid a possible effect of the position of each well on the mycelial growth. The experiment was conducted twice. After 5 days of incubation, the largest and smallest diameters of the colonies of each *Verticillium* isolate were measured using a ruler and the mean data represented total growth (mm).

The potential of FO12-produced VOCs to reduce the viability of the microsclerotia produced by *V. longisporum* ELV25 and *V. dahliae* ELV22 was separately evaluated. For that purpose, microsclerotia from both *Verticillium* species were obtained as described in Varo et al. (2016a). The microsclerotia were produced in Czapek Dox liquid culture (Sigma-Aldrich) prepared in Erlenmeyer flask of 300-ml capacity each containing 100 mL of the medium. For each *Verticillium* isolate, a conidial suspension (10^6^ conidia mL^–1^) obtained from 7-day-old colony was used to inoculate the flasks. Liquid cultures were incubated at room temperature in an orbital shaker (Grant bio PSU-20i, Grant Instruments, Cambridge, United Kingdom) at 90 rpm for 28 days in the dark. The obtained microsclerotia suspensions were homogenized by using a FastPrep-24 device (MP Biomedicals, Santa Ana, CA, United States) for 8 s at 4 m s^–1^. Subsequently, microsclerotia suspensions were adjusted with sterile distilled water by using a hemocytometer to 10^6^ microsclerotia mL^–1^. The experiment was carried out with a modified “Two Clamp VOCs Assay.” For each *Verticillium* isolate, wells of one 6-well plate were filled with 300 μL of the microsclerotia suspension and dried in sterile conditions until the remaining water was evaporated. The initial number of microsclerotia per well was 3 × 10^5^. One 6-well plate with mycelial plugs (3 mm ø) of *F. oxysporum* FO12 was placed opposite to the plate with the microsclerotia, separated by the perforated silicon foil and fixed with two clamps. The assay was performed in six replicates (six wells for each *Verticillium* isolate and FO12 combination) and the experiment was conducted twice. Additionally, six wells with microsclerotia connected to a plate only with PDA were used as a control. The plates were incubated for 7 days at room temperature. After the period of incubation, the microsclerotia from each well were recovered with 700 μL of sterile distilled water in 1 ml tubes. The viability of the microsclerotia was tested by plating several serial dilutions of 100 μL of each recovered microsclerotia suspension on PDA plates incubated for 3 days at room temperature in the dark. After 3 days of incubation, the number of *Verticillium* colonies per PDA plate was counted in order to obtain the total number of viable microsclerotia (CFU). The average number of CFU per well was obtained from three PDA plates, resulting a total of 18 PDA plates for each treatment combination (3 PDA plates/well × 6 wells/treatment combination).

### Analysis of VOCs Produced by *F. oxysporum* FO12

The identification of FO12-emitted VOCs was conducted in GC-MS headspace solid phase micro extraction experiments with minor adaptions as described by Cernava et al. (2015a). For samples preparation, one mycelial plug (3 mm ø) of FO12 was transferred into a 20 mL headspace vial (75.5 mm × 22.5 mm; Chromtech, Idstein, Germany) previously filled with 8 mL of PDA. In order to test the VOCs produced by FO12 in presence of the pathogens, additional vials with mycelial plugs of ELV25 and ELV22 were prepared. Vials with the BCA were co-incubated together with those with *V. dahliae* or *V. longisporum* in a sterile glass jar (0.5 L) hermetically closed in order to exchange their VOCs without direct contact with one another. FO12 vials incubated without the presence of the pathogens were added as a control. All vials were incubated at room temperature for 3 days and the glass jars were opened every 12 h to ensure aerobic conditions. Following 3 days of incubation, vials were aerated under sterile conditions for 2 h to avoid the presence of VOCs produced by *Verticillium* isolates in the vials inoculated with FO12. Subsequently, vials were separately sealed with adequate crimp seals and incubated for additional 3 h for VOCs accumulation. Three replicated vials were used per each pathogen/FO12 combination. Vials containing only PDA were analyzed under the same conditions and used to subtract compounds originating from the medium. Identification of the volatile compounds was performed with NIST MS Search 2.2 included in the Software-Package of the NIST 2014 database. Further verification was done by calculation of the Kovats index (KI) followed by comparisons to database entries of NIST Search 2.2 and the entries in the Online Database maintained by NIST^1^.

### Soluble Metabolite Analyses of *F. oxysporum* FO12

The identification of the soluble metabolites from *F. oxysporum* FO12 was carried out as described by Rybakova et al. (2017). *V. dahliae* ELV22 and *F. oxysporum* FO12 were co-incubated in order to exchange their VOCs without direct contact with one another. A petri dish with *V. dahliae* ELV22 was placed on top of the *F. oxysporum* FO12 plate both transferred in the groove by means of a sterile handle just before the incubation and sealed to facilitate the accumulation of VOCs. Plates with FO12 in co-incubation with non-inoculated PDA plates were included as a control. The experiment was conducted in three replicates. Cell lysis was performed by using a FastPrep-24 device (MP Biomedicals, Santa Ana, California, United States) for two times 30 s at 6 m s^–1^ in 90% methanol. The cell-free extract was stored at −70°C. The *F. oxysporum* FO12 metabolite extracts were analyzed with a combined HPLC hybrid quadrupole-orbitrap mass spectrometer (Q Exactive; Thermo Scientific, Bremen, Germany). To separate different metabolites from the cell extracts, an Atlantis dC18, 3 μm, 2.1 mm × 100 mm column (Waters GesmbH, Phenomenex, Vienna, Austria) was used as described by Cernava et al. (2015b). Identification of the soluble compounds was performed with the XCalibur 2.2 and Compound Discoverer 2.1 (Thermo Scientific, Bremen, Germany) and manual comparison of the spectra with corresponding spectra from literature as well as such from mzCloud (HighChem LLC, Bratislava, Slovakia).

### *In situ* Visualization of *F. oxysporum* FO12 in Oilseed Rape

In order to study root colonization of the non-pathogenic *F. oxysporum* strain FO12, a colonization assay was conducted following the modified protocol described by Rybakova et al. (2016). A total of 16 surface-sterilized oilseed rape (*Brassica napus* L. “Traviata H 605886”; KWS Saat Einbeck, Germany) seeds were aseptically placed into two germination pouches (Mega International, Minneapolis, MN, United States) (8 seeds per pouch) previously filled with 15 mL of sterile distilled water. The pouches were placed into sterilized plastic containers and incubated under gnotobiotic conditions in a greenhouse at 22°C and a 12 h photoperiod. After 4 days of incubation, germinated seedlings were inoculated in germination pouches by roots drenching with 200 μL of a conidial suspension (10^6^ conidia mL^–1^) from a 5-day-old colony of the sGFP-labeled *F. oxysporum* FO12-sGFP. After the inoculation, the seedlings were kept in the greenhouse for 14 days at the conditions described above.

For fluorescence microscopy visualization, two oilseed rape seedlings were sampled at 4, 6, 8, 10, 14, and 17 DAI. The root and stem of the seedlings were cut into small pieces with a sterile razor blade. Seedlings samples were additionally stained with calcofluor white (CFW; 1 g/l; Sigma-Aldrich) for improved imaging of host structures. Subsequently, samples were transferred on optical slides. To study colonization patterns a Leica TCS SPE confocal laser scanning microscope (CLSM) (Leica Microsystems, Mannheim, Germany) was used. sGFP and calcofluor staining were sequentially excited with 635 and 405 nm laser beams, respectively. The confocal stacks were acquired with a LeicaACS APO 40 × oil CS objective lens (NA, 1.30) and for each field of view, an appropriate number of optical slices were acquired with a Z-step ranging from 0.15 to 0.5 μm. Laser settings were adjusted to maximize signal to noise ratio of both fluorescent signals (sGFP and CFW). The software Imaris 7.3 (Bitplane, Zurich, Switzerland) was used for imaging and post-processing of the confocal stacks and maximum projections. Additionally, at the end of the experiment, three seedlings were harvested to perform re-isolations to confirm FO12-sGFP colonization. For this purpose, stem and root of each seedling were cut into six small pieces and plated on PDA-hygromycin B. Subsequently, plates were incubated at room temperature for 5 days and positive isolations were recorded.

### Statistical Analysis

Analysis of variance of the mycelial growth (mm), microsclerotia viability (CFU) and abundance of metabolites produced by FO12 were performed according to a completely randomized design. The data from replicated experiments were combined after assessment of the homogeneity of the experimental error variances by the *F-*test. Furthermore, data were tested for normality, homogeneity of variances, and residual patterns, which proved their suitability for the statistical analysis. When analysis of variance showed significant differences among treatments, means were compared according to Fisher’s protected least significant differences (LSD) test at *P* = 0.05. All data of this study were analyzed using Statistix 10 (Analytical Software, Tallahassee, FL, United States).

## Results

### Effect of *F. oxysporum* FO12 VOCs on Mycelial Growth of *V. longisporum* and *V. dahliae*

**FIGURE 1:**
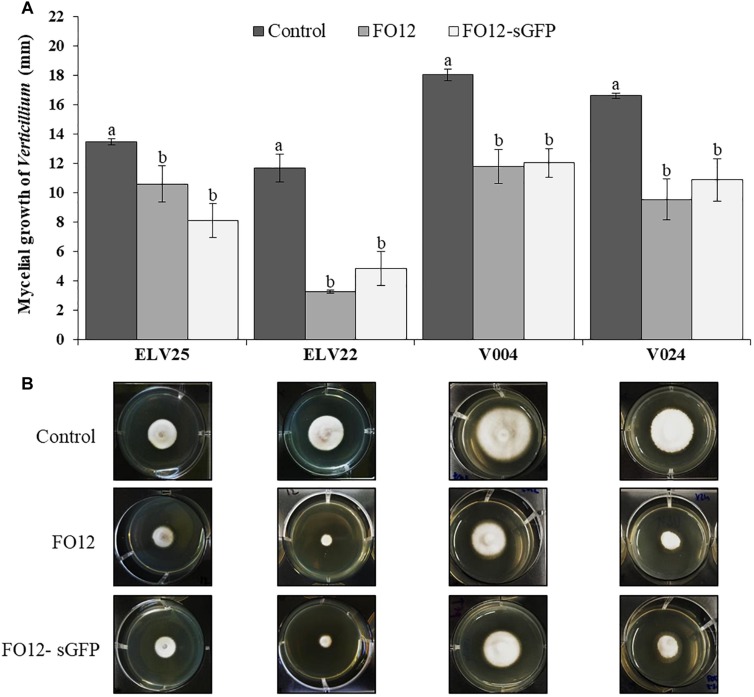
Effect of volatile organic compounds (VOCs) from the non-pathogenic *F. oxysporum* strain FO12 and from the GFP-labeled FO12 (FO12-sGFP) against the mycelial growth of *V. longisporum* ELV25 and *V. dahliae* isolates ELV22, V004 and V024 **(A)**. For each isolate, columns represent the means of 12 replicates per treatment. Vertical lines in each column are the standard error of the mean. For each *Verticillium* isolate, means in a column followed by different letters are significantly different according to Fisher’s protected least significant differences (LSD) test at *P* = 0.05. **(B)** Pictures below illustrate the mycelial growth of each *Verticillium* isolate according to the different treatments.

The effect of VOCs emitted by *F. oxysporum* FO12 and FO12-sGFP on mycelial growth of different *Verticillium* isolates was assessed in co-incubation experiments. VOCs produced by both FO12 and FO12-sGFP reduced the mycelial growth of the phytopathogenic fungi when they shared the same headspace. Mycelial growth of *V. longisporum* ELV25 was significantly reduced (*P* = 0.0029) with a final growth diameter of 10.5, 8.1, and 13.5 mm for FO12, FO12-sGFP and control treatments, respectively (Figure 1A). Moreover, VOCs emitted by FO12 and FO12-sGFP were also able to significantly reduce the mycelial growth of *V. dahliae* isolates ELV22 (*P* < 0.0001), V004 (*P* = 0.0003), and V024 (*P* = 0.0016) in comparison with their respective controls. The final growth diameter for the ELV22 strain was 3.25, 4.83, and 11.67 mm; for strain V004 it was 11.79, 12.04, and 18.04 mm; and for strain V024, it was 9.54, 10.88, and 16.63 mm for FO12, FO12-sGFP and control treatments, respectively (Figure 1A). The VOCs-mediated reduction of mycelial growth by the *F. oxysporum* strain and the sGFP mutant was more pronounced in *V. dahliae* isolates than in *V. longisporum* ELV25 (Figure 1B). The effectiveness of VOCs emitted by *F. oxysporum* FO12 and FO12-sGFP against mycelial growth of *Verticillium* isolates was similar and no significant differences were found between the strains.

### Effect of VOCs on Microsclerotia of *V. longisporum* and *V. dahliae*

**FIGURE 2:**
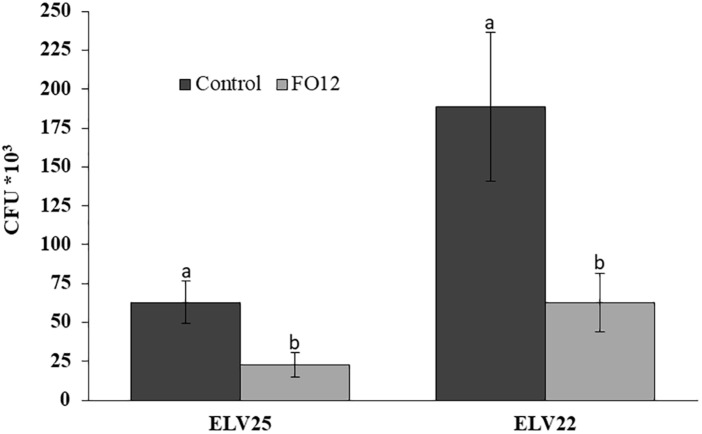
Effect of volatile organic compounds (VOCs) from the non-pathogenic *F. oxysporum* strain FO12 on microsclerotia viability of *V. longisporum* ELV25 and *V. dahliae* ELV22. For each isolate, columns represent the means of 12 replicates per treatment. Vertical lines in each column are the standard error of the mean. For each *Verticillium* isolate, means in a column followed by a different letter are significantly different according to Fisher’s protected least significant differences (LSD) test at *P* = 0.05.

A modified version of the “Two Clamp VOCs Assay” (Cernava et al., 2015a) was used to evaluate the efficacy of the VOCs from the non-pathogenic FO12 strain to reduce the viability of microsclerotia. After exposure of *V. longisporum* ELV25 and *V. dahliae* ELV22 microsclerotia to FO12-emitted VOCs, their viability was significantly decreased. The incubation of recovered microsclerotia on PDA after the treatment resulted in lower CFU numbers for treated samples. In detail, the exposure resulted in a significant reduction of the viability (22.7^*^10^3^ CFU; *P* = 0.0252) of *V. longisporum* ELV25 in comparison with the control (62.9^*^10^3^ CFU) (Figure 2). VOCs produced by FO12 were also able to significantly reduce the viability of microsclerotia from *V. dahliae* ELV22 (62.8^*^10^3^ CFU; *P* = 0.0282) in comparison with the control (188.5^*^10^3^ CFU) (Figure 2).

### Identification of VOCs Produced by *F. oxysporum* FO12

A total of 21 VOCs produced by FO12 were identified by means of GC-MS analysis based on their mass spectra (Table 1). These VOCs belong to different chemical groups and included terpenes, alcohols, esters, cyclic carbon compounds, as well as alkanes. VOCs belonging to the terpene group were the most abundant ones (6/21) and distinct compounds (cedr-8-ene, cembrene, and β-acorenol) were produced both when FO12 was incubated either alone or after the exposure to *Verticillium*. Contrarily, β-cedrene was produced only after exposure to *V. longisporum.* Different alcohols and esters were also abundant among the identified compounds (4/21). They were constantly produced by FO12 and only 2-methyl-1-propanol (alcohol) and 2-methylbutyl acetate (ester) were produced specifically after exposure to both *Verticillium* species or of *V. dahliae*, respectively (Table 1). We identified three volatiles which included cyclic carbons in their structure as main chemical group; two of them were produced only when FO12 was exposed to both *Verticillium* species and the other one was constantly emitted by FO12 (Table 1). In terms of alkanes, tridecane and hexane, 2, 3,-dimethyl were detected and both were constantly produced by FO12. Finally, volatiles assigned to the aromatic compounds group, such as 1-ethyl-4-methoxybenzene and pyrocatechol, were emitted by FO12 constantly or after exposure to the two *Verticillium* species, respectively.

**TABLE 1:** GC-MS headspace SPME identification of relevant VOCs produced by the non-pathogenic *F. oxysporum* FO12 alone and during co-incubation with *V. longisporum* ELV25 or *V. dahliae* ELV22.

**Predicted compound^a^**	**KI^b^**	**Match^c^**	**Predicted funtion^d^**
**VOCs constantly produced by FO12**			
3-methyl-1-butanol	736	966	Antifungal (De Souza et al., 2018)
2-methyl-1-butanol	739	934	Antifungal (Raza et al., 2015)
β-acorenol	1649	922	Antibacterial (Albay et al., 2009)
Ethyl acetate	612	873	Antifungal (Toffano et al., 2017)
1-hexanol	868	928	PGP (Splivallo et al., 2007)
Cedr-8-ene	1411	936	N.a.
3-methylbutyl acetate	876	907	PGP (Amavizca et al., 2017)
Isobutyl acetate	771	838	N.a.
1-ethyl-4-methoxybenzene	1110	887	N.a.
Tridecane	1300	790	PGP (Amavizca et al., 2017)
1,1,2b,6-tetramethyl-2,2a,2b,3,4,6a,7,7a-octahydro-1H-cyclobuta[a]indene	1330	843	N.a.
Hexane, 2,3-dimethyl	760	806	N.a.
**VOCs produced by FO12 after exposure to *V. longisporum* or *V. dahliae***			
(1R,4R,5S)-1-isopropenyl-4,8-dimethylspiro[4.5]dec-7-ene	1475	917	N.a.
2-methyl-1-propanol	625	845	Antifungal (Stotzky et al., 1976)
Pyrocatechol	2020	937	N.a.
Cembrene	1939	933	N.a.
Aristol-1-ene	1453	862	N.a.
(4R,5R)-1-isopropylidene-4,8-dimethylspiro[4.5]dec-7-ene	1515	906	N.a.
Alloaromadendrene	1461	909	N.a.
**VOCs produced by FO12 only after exposure to *V. longisporum***			
β-cedrene	1421	921	N.a.
**VOCs produced by FO12 only after exposure to *V. dahliae***			
2-methylbutyl acetate	880	887	Nematicidal (Terra et al., 2018)

*^*a*^Compound names according to International Union of Pure and Applied Chemistry (IUPAC). ^*b*^Kovats index (KI) of the compounds was calculated with an alkane series. ^*c*^Match index: Only substances with match index with the NIST MS Search 2.2 included in the Software-Package of the NIST 2014 database over 750 were considered. ^*d*^Predicted function of the compounds according to referenced literature. N.a., not available (unknown function). PGP, plant growth promotion.*

### Changes in Soluble Metabolites of *F. oxysporum* FO12 After Exposure to *V. dahliae* VOCs

The composition of soluble metabolites produced by *F. oxysporum* FO12 grown in the presence of VOCs produced by *V. dahliae* ELV22 was assessed by means of high-resolution LC-MS analyses. Relative abundance of the 26 compounds produced by FO12 was affected in the presence of the pathogen. These metabolites are involved in distinct pathways and have different metabolic functions as shown in Table 2. The interaction with *V. dahliae* mostly affected the pathways associated with amino acids metabolism. Within this group, some compounds as pantothenic acid related to the metabolism and synthesis of carbohydrates, proteins, and fats showed a significant upregulation (2.42 fold). In contrast, L-ergothioneine showed a significant downregulation (−2.40 fold) (Table 2). The abundance of metabolites associated with carbohydrate metabolism pathways was also highly affected by the interaction with the pathogen. Thus, gluconic acid and alpha,alpha-trehalose showed a downregulation by ELV22 VOCs (−2.22 and −3.24 fold, respectively). Beauvericin was also significantly downregulated (−1.89 fold) when FO12 was co-incubated with the pathogen (Table 2). Finally, we also detected a significant decrease in indole-3-lactic acid production (−1.95 fold), following exposure to ELV22.

**TABLE 2:** Effects of exposure to *V. dahliae* ELV22 on *F. oxysporum* FO12 metabolism detected by LC-MS.

**Predicted metabolite^a^**	**Fold change^b^**	**Metabolic function^c^**
		**Amino acid metabolism**
N-acetyl-L-methionine	28.88	Cysteine and methionine metabolism
N-acetyl-L-phenylalanine	7.15^*^	Phenylalanine metabolism
N-acetylvaline	6.85	Valine, leucine and isoleucine degradation
N-acetylornithine	4.74	Arginine biosynthesis
4-acetamidobutanoic acid	2.48^*^	Arginine and proline metabolism
Pantothenic acid	2.42^*^	Beta-Alanine metabolism
N-acetyl-DL-tryptophan	2.34^*^	Tryptophan metabolism
L-glutathione (reduced)	1.53^*^	Glutathione metabolism
2-isopropylmalic acid	–1.49	Valine, leucine and isoleucine degradation
L-glutamic acid	–1.98	Arginine biosynthesis
L-ergothioneine	−2.40^*^	Histidine metabolism
L-saccharopine	−2.56^*^	Lysine biosynthesis
L-aspartic acid	−2.57^*^	Arginine biosynthesis
L-glutathione oxidized	–13.70	Glutathione metabolism
		Carbohydrate metabolism
D-(+)-maltose	1.93	Starch and sucrose metabolism
Gluconic acid	–2.22	Pentose phosphate pathway
Alpha,alpha-trehalose	−3.24^*^	Starch and sucrose metabolism
N-acetyl-D-galactosamine	−3.61^*^	Amino sugar and nucleotide sugar metabolism
α-D-mannose 1-phosphate	−4.61^*^	Fructose and mannose metabolism
α-D-glucose-1,6-bisphosphate	−8.69^*^	Starch and sucrose metabolism
		**Lipid metabolism**
(±)12(13)-DIHOME	2.79	Linoleic acid metabolism
(15Z)-9,12,13-trihydroxy-15-octadecenoic acid	−1.85^*^	Fatty acid biosynthesis
		**Nucleotide metabolism**
Uric acid	8.42	Purine metabolism
		**Energy metabolism**
Flavin mononucleotide (FMN)	6.51^*^	Oxidative phosphorylation
		**Chemical structure transformation maps**
Beauvericin	−1.86^*^	Fungal toxin
Indole-3-lactic acid	−1.95^*^	Biosynthesis of plant hormones

^*a*^Only substances that were up- or down-regulated by *V. dahliae* ELV22 VOCs are shown. ^*b*^Abundance ratio between compounds from FO12 in co-incubation with ELV22 and from unexposed controls. Positive and negatives values correspond to upregulated and downregulated substances by *V. dahliae* ELV22 VOCs, respectively. Only metabolites with an up- or downregulation higher than 1.5 or lower than –1.5 were added. Values followed by an asterisk (^*^) indicate that the up- or downregulation was statistically significant according to Fisher’s protected least significant differences (LSD) test at *P* = 0.05. ^*c*^Metabolic pathways assigned by Kyoto Encyclopedia of Genes and Genomes. Terms in bold indicate metabolic pathway groups. N.a., not available indicates that the metabolic pathway could not be found.

### Root Colonization of FO12 in Oil Seed Rape

**FIGURE 3:**
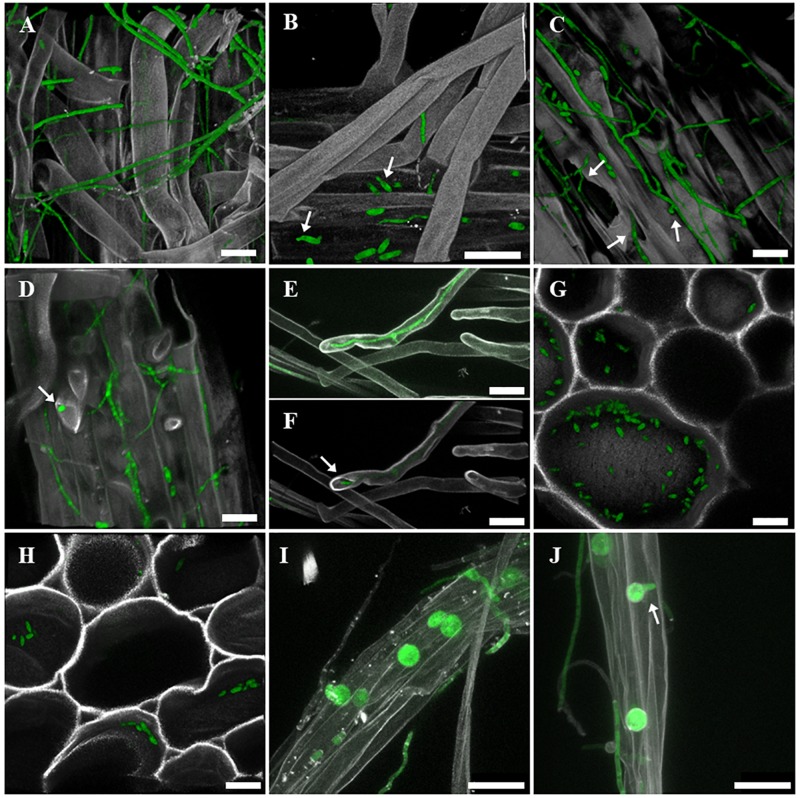
Confocal laser scanning microscopy (CLSM) micrographs showing the colonization pattern of oilseed roots and stem by the strain FO12-sGFP. Green, fungus; gray, host tissues stained with white calcofluor. **(A)** Extensive root hairs colonization by FO12-sGFP, 10 days after inoculation (DAI). **(B)** Germinating microconidia (indicated by arrows) attached to the main root tissue at 6 DAI. **(C)** Extensive main root colonization and root infection by FO12-sGFP toward micro-injuries and by appressorium 8 DAI (arrows indicate micro-injuries and appressorium for FO12-sGFP penetration). **(D,E,F)** Endophytic behavior of FO12-sGFP growing inside root hairs at 6 DAI (arrows indicate the detail of a hypha inside the root hair). **(G,H)** Conidial spread of FO12-sGFP toward the stem, 17 DAI. **(I,J)** Embedded chlamydospores in root hairs bundles at 14 DAI (arrow indicates a germinating chlamydospore). Scale bars: 25 μm.

After root inoculation of oilseed seedlings with the sGFP-labeled *F. oxysporum* FO12-sGFP strain, roots and stem of the seedlings were sampled for CLSM visualization. FO12-sGFP was able to extensively colonize the roots of oilseed seedlings. sGFP-labeled hyphae were observed growing between root hairs of oilseed seedlings (Figure 3A) as well as attached to the surface of the main root following preferably the root growth direction (Figures 3C,D). Germinating microconidia attached to the main root surface were observed at 6 DAI (Figure 3B). Several infection points were observed at 8 DAI where FO12-sGFP was able to infect the seedlings. Figure 3C shows several micro-injuries on the root surface by which hyphae were directly infecting the plant. Formation of appressoria-like structures on the root surface was also observed as an alternative way to infect the plant. Appressoria were preferably formed in the intercellular space of the main root surface (Figure 3C). Confocal microscopy confirmed the endophytic lifestyle of this strain, since hyphae of FO12-sGFP were found growing inside roots hairs at 6 DAI (Figures 3D–F). Figure 3F is a 3D reconstruction of Figure 3E confirming that the fluorescent hypha was growing within a root hair. The spread of microconidia of FO12-sGFP along the xylem vessels of the oilseed stem was observed at 17 DAI, but no hyphal colonization of the stem was found (Figures 3G,H). At 14 DAI, the presence of embedded chlamydospores of FO12-sGFP in root hairs bundles was detected (Figures 3I,J). Chlamydospores were able to germinate in order to continue the root colonization (Figure 3J). Additionally, FO12-sGFP was consistently re-isolated from the stem and root of seedlings harvested at the end of the experiment.

## Discussion

In the present study, new insights related to the interaction between the non-pathogenic *F. oxysporum* strain FO12 and different pathogenic Verticillium species were obtained. We have identified VOCs, which could play an important role in the antagonistic interactions between the two fungi. Moreover, rhizosphere colonization patterns of the potential competitors showed that they occupy the same niche within the plant, which suggest competition between them. The exposure of *V. longisporum* and *V. dahliae* to VOCs from FO12 resulted in a significant inhibition of mycelial growth in both pathogens. These results indicate that the antagonistic effect reported by Varo et al. (2016b) when *V. dahliae* was confronted with FO12 in dual cultures, was at least partially due to the production of volatile compounds with inhibitory activity against the pathogen. The effect of microbial VOCs from various BCAs against pathogenic fungi was addressed in recent studies (e.g., Zhang et al., 2015; Rybakova et al., 2017). The results of the current study are in agreement with those reported by Zhang et al. (2015) in which VOCs produced by the non-pathogenic strain CanR-46 of *F. oxysporum* inhibited the growth of different phytopathogenic fungi, including *V. dahliae*. In addition, we found that VOCs produced by FO12 were able to significantly reduce the viability of microsclerotia of both *V. longisporum* and *V. dahliae*. The effectiveness of FO12 in reducing inoculum density of *V. dahliae* in naturally infested soils was also recently reported (Varo et al., 2016b). It was shown that FO12 was able to completely inhibit the viability of microsclerotia when it was applied to naturally infested soils. Although a total reduction of microsclerotia viability with the VOCs assay was not achieved, our results suggest that a high proportion of the observed inhibition effect can be attributed to VOCs produced by FO12. In addition, after exposure with VOCs, a fraction of microsclerotia from both pathogens was unable to germinate on PDA plates, confirming the fungitoxic effect of the VOCs produced by FO12.

Interestingly, some of the identified VOCs produced by *F. oxysporum* FO12 were short-chain alcohols with known antifungal properties. The biocontrol activity of 3-methyl-1-butanol and 2-methyl-1-butanol has been confirmed in previous studies. Several compounds belonging to the chemical group of alcohols have been reported to have antifungal activity including 3-methyl-1-butanol (De Souza et al., 2018), 2-methyl-1-butanol (Raza et al., 2015), and 2-methyl-1-propanol (Stotzky et al., 1976). De Souza et al. (2018) reported the capability of *Saccharomyces cerevisiae* to produce 3-methyl-1-butanol and 2-methyl-1-butanol which, among others VOCs, were able to significantly reduce the growth of *Penicillium digitatum*. Additionally, Lopes et al. (2015) observed a total inhibition of *Colletotrichum gloeosporioides* and *C. acutatum* by 3-methyl-1-butanol and 2-methyl-1-butanol produced by *S. cerevisiae*. Interestingly, also distinct *Verticillium* species were shown to produce both of these alcohols (Li et al., 2018). It remains to be elucidated if producers of these compounds are less affected by inhibitory effects of *F. oxysporum* FO12. The inhibitory effects of alcohols seems to affect the organization and stability of the lipid bilayer from the plasma membrane (Fialho et al., 2010; Toffano et al., 2017). Within the alcohol group, 1-hexanol, a commonly produced fungal VOC, was reported to reduce *Arabidopsis thaliana* growth (Splivallo et al., 2007). Among terpenes, β-acorenol is known for its antibacterial activity (Albay et al., 2009). A broad range of biological functions have been found among esters compounds, such as ethyl acetate with antifungal activity against *Sclerotinia sclerotiorum* when it is produced by *S. cerevisiae* (Toffano et al., 2017). The ester 3-methylbutyl acetate has known plant growth promotion activity, enhancing the performance of the microalga *Chlorella sorokiniana* (Amavizca et al., 2017). Additionally, within the ester group, 2-methylbutyl acetate was reported to show nematicidal activity (Terra et al., 2018). Finally, among alkanes, tridecane is also known for its plant growth promotion activity (Amavizca et al., 2017). Although some of the VOCs found have been reported for antimicrobial activity, the biological function of compounds such as cembrene, alloaromadendrene or pyrocatechol, among others, and volatiles belonging to the cyclic carbon compounds group, remain unknown. The identification of bioactive volatile compounds in this study supports the hypothesis that the antagonistic effect of FO12 on mycelial growth and microsclerotia viability of *V. dahliae* is mainly due to the production of VOCs with antifungal activity. Most of the VOCs found in this study with biocontrol activity were continuously produced by FO12, which indicates that the production of antifungal volatiles from FO12 could be a rather unspecific strategy of the BCA to shield of competitors.

Recently, Rybakova et al. (2017) reported that microorganisms are able to mutually regulate their metabolism by means of an interchange of aerial signals such as VOCs. This communication between microorganisms may induce a differential metabolic performance in order to enhance or reduce the production of specific soluble metabolites to guarantee the recipient’s survival in the environment. Compounds involved in the metabolism of amino acids, carbohydrates, lipids, nucleotide, energy or other chemical structures can be responsible for an aerial dialogue between microorganisms. This facilitates broad adaptability of the interacting microorganisms to biotic stress. In this study, regulation of FO12’s metabolism was observed when the production of several soluble metabolites was up- or down-regulated in presence of *V. dahliae* ELV22. Among the metabolites involved in amino acid metabolism, ergothioneine, which showed a significant downregulation, is biosynthesized exclusively by some fungi and mycobacteria and it has a role as a physiologic cytoprotectant (Paul and Snyder, 2009). The downregulation of alpha,alpha-trehalose, a metabolite used in starch and sucrose metabolism, might indicate that the BCA is improving the stress-resistance of its cells (Wyatt et al., 2015) prior to the interaction with the pathogen. In addition, we found several metabolites with antifungal activity such as gluconic acid (Kaur et al., 2006) and beauvericin (Wang and Xu, 2012), both showing a downregulation in the presence of the pathogen. Some microorganisms are known for the biosynthesis of plant hormones like auxins (Liu et al., 2016), in this context, we also detected downregulation of indole-3-lactic acid, a metabolite involved in the biosynthesis of plant hormones as auxin (Sardar and Kempken, 2018) when FO12 was interacting with the phytopathogen. Although our data indicate an extensive regulation of FO12 metabolic pathways during its interaction with the pathogen, further research is needed in terms of how the regulation of FO12 metabolism interferes with the antagonistic effect of this BCA against *V. dahliae*.

Root colonization patterns of FO12 by means of CLSM showed the entire process of colonization, beginning with conidial germination on the root surface until the formation of resting structures (chlamydospores). The extensive root surface colonization by FO12 was consistent with the root colonization patterns of the non-pathogenic isolate Fo47 of *F. oxysporum* in pepper (Veloso et al., 2016). No preferential growth along the intercellular junctions was observed. This is contrary to the observation reported by Pantelides et al. (2009) in which strain F2 grew attached to intercellular space on eggplant roots. This observation confirms the hypothesis by the same author that non-pathogenic *F. oxysporum* strains have their own colonization pattern, as before suggested by Steinberg et al. (1999). After conidial germination, FO12 was able to infect the roots through micro-injuries and appressoria formation on the root surface. This observation indicates that FO12 has similar infection sites preferences as *V. dahliae* as reported by Veloso et al. (2016) after visualization of the interaction between Fo47 and *V. dahliae* in pepper rhizosphere. Thereby competition for space and infection points could play an essential role in the control of VWO as observed by Varo et al. (2016b). Moreover, the endophytic behavior of FO12 and conidial spread along the vascular system was confirmed. The systemic plant colonization by the BCA following the xylematic flux is the same strategy used by *V. dahliae*, although no symptomatic plants by FO12 were observed. One of the most interesting observations conducted in this study was the formation of chlamydospores embedded in root hairs bundles. The capability of FO12 to form chlamydospores is considered an important trait of this BCA to ensure a long-term survival and antagonistic effect against the pathogen under field conditions. Moreover, the capacity to form resting structures facilitates the development of future commercial formulations.

Various traits of FO12 are in agreement with those proposed by Deketelaere et al. (2017) that are desirable for a promising BCA toward *Verticillium* because (i) the produced VOCs affect microsclerotia and mycelia, (ii) colonize the same ecological niche than the pathogen, and (iii) compete with the pathogen. Understanding the ecology, interactions, and evolution of microbial key players in agricultural microbiomes will have a great potential for food security and safety. In contrast to the pathogenic effects of various species, recent research results indicate a natural function of *Verticillium* for plants: VOCs for auxin signaling and ripening of plants (Li et al., 2018) and their aroma production (Landa et al., 2019). These important findings should be considered for upcoming plant protection strategies as well in order to maintain functioning of non-pathogenic species.

## Data Availability

All datasets generated for this study are included in the manuscript and/or the supplementary files.

## Author Contributions

TC, FL-E, AT, and GB designed the study. DT and AM-A carried out the transformation with sGFP gene under the supervision of AD. AM-A carried out the VOCs, GC-MS, soluble metabolites, colonization experiments, analyzed the GC-MS data, and subjected the VOCs experiments data to statistical analyses. AS conducted the soluble metabolites data analyses. TC and DT contributed to the writing of the VOCs and transformation-related parts of the final manuscript, respectively. AM-A and TC wrote the final version of the manuscript. GB, FL-E, AD, and AT reviewed the final version of the manuscript. All authors read and approved the final version of the manuscript.

## Conflict of Interest Statement

The authors declare that the research was conducted in the absence of any commercial or financial relationships that could be construed as a potential conflict of interest.

## Footnotes

1 http://webbook.nist.gov/

## Abbreviations

BCA(s): biological control agent(s)
CFU: colony forming units
CLSM: confocal laser scanning microscopy
DAI: days after inoculation
GC-MS: gas chromatography-mass spectrometry
sGFP: super-folder green fluorescent protein
VOCs: volatile organic compounds
VWO: verticillium wilt of olive.
